# Leveraging Machine Learning to Advance Alcohol Research: Current Applications, Challenges, and Opportunities

**DOI:** 10.35946/arcr.v46.1.03

**Published:** 2026-06-05

**Authors:** Qingyu Zhao, Kilian M. Pohl

**Affiliations:** 1 Department of Radiology, Weill Cornell Medicine, New York, New York; 2 Department of Psychiatry & Behavioral Sciences, Stanford University, Stanford, California; 3 Department of Electrical Engineering, Stanford University, Stanford, California

**Keywords:** alcohol, machine learning, predictive

## Abstract

**PURPOSE:**

The review surveys the type of machine learning approaches currently used in the alcohol literature, reviews challenges in applying machine learning tools to alcohol data, and explores how overcoming these challenges could advance personalized medicine for alcohol use disorder (AUD).

**SEARCH METHODS:**

The authors conducted a search of publications on PubMed, ScienceDirect, and EBSCO Academic Search Premier published from 2015 to April 15, 2025, for articles that used machine learning to analyze alcohol-related outcomes. Search terms were (“drinking” OR “alcohol”) AND (“machine learning” OR “deep learning” OR “predict” OR “classify”) in the title or abstract.

**SEARCH RESULTS:**

The search returned 2,618 manuscripts. Keeping those that predicted alcohol-related outcomes and excluding those that merely used alcohol as a predictor for other outcomes reduced the selection to 567 manuscripts. A final manual selection resulted in 110 original peer-reviewed human research studies that primarily analyzed alcohol consumption behaviors and tested their models on data that they were not trained on.

**DISCUSSION AND CONCLUSIONS:**

Predictions focused on alcohol consumption or AUD diagnosis in cohorts with a mean age of 50 years or younger (i.e., when long-term drinking behaviors are being or have been established). Most studies confined the data-driven searches to a single modality and relied on conventional machine learning approaches, which tended to produce accurate and transparent predictions on the relatively small datasets typically collected by AUD studies. The small number of available samples was the most common limitation mentioned by the reviewed articles. Investigators also wished for machine learning models to provide insights about causality. Gaining these insights will be essential to improve diagnosis and treatment of AUD, for which the field must foster multidisciplinary research teams to build rigorous and trustworthy machine learning models and quantitative benchmarks that can capture the multifaceted nature of alcohol use and its comorbidities.

**KEY TAKEAWAYS**
- Alcohol-related publications using machine learning almost exclusively relied on conventional techniques, whereas current public discourse emphasizes state-of-the-art models.
- A majority of models predicted alcohol consumption or alcohol use disorder (AUD) diagnosis, which is generally easier to forecast than, for example, disorder or treatment outcome.
- Only 40% of models utilized multimodal data, which is needed for encoding the complexity of AUD and related clinical outcomes.
- Addressing the complexity of AUD requires creating machine learning models and quantitative benchmarks that accurately capture the multifaceted nature of alcohol use and its comorbidities.

Alcohol use disorder (AUD) currently affects 27.9 million Americans^1^ and costs the United States more than $250 billion annually.^2^ It is the fourth leading preventable cause of death,^3^ underscoring the urgent need for effective strategies to understand, predict, and prevent alcohol misuse. At the mechanistic level, AUD arises from complex interactions between neural, genetic, and environmental factors that collectively influence vulnerability and resilience to alcohol’s effects. Genetic and epigenetic factors account for 40% to 60% of a person’s addiction risk,^4^,^5^ although psychological and environmental factors (such as family history,^6^,^7^ traumatic events,^8^ peer pressure,^9^,^10^ other drug use,^11^,^12^ and externalizing behaviors^13^,^14^) also play crucial roles in shaping drinking behaviors. However, findings about these predictive factors are often inconsistent or weak: many individuals without known risk factors develop AUD,^15^ whereas at-risk individuals remain resilient^16^ (e.g., approximately 50% of maltreated youth^17^). Equally pressing is the challenge of predicting treatment response and long-term abstinence. Although pharmacological and behavioral interventions are available, only 7.9% of people with AUD receive alcohol use treatment in the United States,^18^ and of those, only 16% achieve abstinence.^19^

One major hurdle in alcohol research has been the predominant reliance on fragmented, small-scale datasets unable to reveal the complex and heterogeneous nature of AUD.^20^,^21^ The need for larger heterogeneous data sets for AUD is recognized by funding agencies.^22^ Since 2012, the National Institute of Alcohol Abuse and Alcoholism has funded the National Consortium on Alcohol and Neurodevelopment in Adolescence (NCANDA) study^23^ to annually collect brain magnetic resonance imaging (MRI) data, neuropsychology testing, alcohol use, and related data of 831 individuals who were age 12 to 21 at baseline. NCANDA was the first to report on in vivo disruption due to alcohol of white matter microstructural development during adolescence.^24^ An even larger sample (more than 11,000 children age 9 to 10 years at baseline) has been recruited by the Adolescent Brain Cognitive Development (ABCD) Study,^25^ which is tracking brain development, behavior, and environmental changes to gain insights into how early life factors shape substance use and mental health outcomes. Over three decades, the Collaborative Studies on the Genetics of Alcoholism (COGA) has collected phenotypic data^26^ from nearly 18,000 individuals ages 7 to 97 across more than 2,200 families, as well as DNA and electrophysiological measures on a large subset. The longitudinal data set provides a unique opportunity for researchers to identify genes influencing the risk of AUD and related outcomes. Another key effort is the Monitoring the Future (MTF) study—an ongoing, nationally representative study that has been surveying more than 25,000 8th, 10th, and 12th grade students^27^ and approximately 20,000 adults ages 19 to 65 each year since 1973.^28^ By capturing longitudinal behavioral and attitudinal changes, MTF offers a unique resource for understanding population-level patterns and risk factors for substance use. Finally, aiming to advance personalized medicine, the All of Us Research Program^29^ is planning to collect multimodal data (including genomic profiles, electronic health records, and environmental exposure) from at least 1 million people in the United States to discern how individual differences in lifestyle, environment, and biology affect health outcomes, such as the relationship between risky drinking behavior and cancer diagnosis.^30^

In addition to increasing sample sizes, alcohol studies have expanded the scope and diversity of data collected from each participant to enable more precise characterization of them. Current studies increasingly integrate more comprehensive self-reports,^31^ medical records,^32^ genetic profiling,^33^ neuropsychological testing,^34^ and imaging.^35^ The advent of wearable technologies and ubiquitous mobile devices (such as smartphones^36^ and biosensors measuring physical activity^37^ and intoxication^38^) has made it possible to conduct ecological momentary assessments.^39^,^40^ This surge in data volume introduces substantial computational and methodological challenges, regardless of cohort size.

The emergence of larger and more diverse data sets calls for a transformation in fundamental analytical approaches. Alcohol research has traditionally relied on hypothesis-driven analyses, which involve preselecting a narrow set of variables^41^,^42^ and applying univariate regression models at the group level to test pairwise associations. Although conceptually straightforward and statistically interpretable, this approach fragments understanding into siloed single factors that each explain only a small fraction of variance in alcohol outcomes, failing to capture the intricate multivariate interactions among behavioral traits, cognitive functions, environmental influences, and neural mechanisms that underlie AUD.^43^,^44^ Furthermore, the findings only reflect group-level trends that generally do not translate to individual-level insights.^45^

A promising alternative is machine learning (Figure 1), which is designed to translate complex, multimodal data into predictions on an individual basis.^46^ Machine learning is a branch of artificial intelligence (AI) that enables computers to identify interactions among measurements (i.e., patterns) predictive of the outcome directly from data rather than relying on predefined statistical formulas or human-crafted rules. Typically, a machine learning model needs to be trained on a large number of samples, where each sample is described by a set of features (predictor variables) and a target outcome variable of interest (e.g., diagnosis, symptom severity, drinking level, or treatment response). In alcohol research, predictors may span multiple domains, including neuroimaging (e.g., functional connectivity, cortical thickness, regional volume, or microstructural integrity), behavioral and cognitive traits (e.g., measures of impulsivity, decision-making, or sensation seeking), environmental exposures (e.g., peer drinking, family history, or neighborhood stressors), and genetic or physiological markers (e.g., polygenic risk scores, heart rate, sleep disturbance). The goal of the model is to integrate information across these domains to generate a single prediction of the chosen target outcome specific to each individual.

During training, the model learns (or is trained) by iteratively adjusting its internal parameters to minimize the prediction error, which is the difference between actual and predicted outcome of the training samples. Once this error is minimized, the parameterized model is evaluated on a set of test samples (i.e., participants and their data not used for training). This evaluation assesses the degree to which the model generalizes to new individuals. Common evaluation metrics^47^ include accuracy (i.e., quantifying the proportion of correct predictions) and the area under the receiver operating characteristic curve (AUC), which measures how well the model discriminates between outcome classes over the full range of decision thresholds. Lastly, one can identify the pattern driving inference, offering insights into potential mechanisms or risk factors underlying alcohol outcomes.

Conventional machine learning models (e.g., random forest, support vector machine [SVM]) typically confine analysis to tabular data, where summary scores are extracted from raw measurements before training the model. For example, brain MRI studies relied on region-level summaries (such as brain volume or cortical thickness) as input to machine learning models, which confined prediction accuracy to the anatomical granularity of the brain regions defined by human experts beforehand.^48^ Removing this constraint necessitates performing predictions directly from raw higher-dimensional data (e.g., all voxels of a 3D brain MRI). This is the domain of deep learning models (Figure 1), a subfield of machine learning that gained traction starting in 2012.^49^ Deep learning models jointly learn to derive measurements (i.e., features) from the raw data and to predict the outcome using large-scale artificial neural networks. Treating feature extraction as part of the optimization process has led to significant improvements in predictive accuracy across numerous domains, fueling growing interest in applying deep learning approaches to data-driven medical research.^50^

This review provides a systematic overview of how machine learning methods have been applied across the landscape of alcohol research over the past decade. Rather than emphasizing the specific constellations of neurobiological or psychological factors identified by machine learning analyses, the review focuses on the methodological trends, data modalities, and modeling choices that characterize current machine learning practice across alcohol-related research. Specifically, the review examines which populations and clinical outcomes have been most frequently targeted, what level of prediction accuracy has been achieved, what types of data have been used to predict outcomes, and how different machine learning models (including deep learning) have been adopted. The review also highlights the challenges associated with applying these methods to AUD studies and discusses how overcoming those challenges could transform diagnosis and treatment from subjective observations to objective, individualized assessments that lead to accurate precision medicine.

## Search Method

In April and July 2025, authors conducted a literature search in PubMed, ScienceDirect, and EBSCO Academic Search Premier for publications that used machine learning to analyze alcohol-related outcomes. Across the three databases, a key word search using (alcohol[Title] OR drinking[Title]) AND (alcohol[Title/Abstract]) AND (“machine learning”[Title/Abstract] OR “deep learning”[Title/Abstract] OR predict[Title/Abstract] OR classify[Title/Abstract]) returned 2,618 manuscripts published from 2015 to April 15, 2025.

A rule-based text-mining Python script (available upon request from the authors) searched for the co-occurrence of prediction-related terms (e.g., predict, model, classify, identify) and alcohol-related terms (e.g., alcohol, drinking, dependence, relapse), while removing articles in which alcohol was a predictor rather than an outcome (e.g., “Alcohol use predicts depression”). This automatic screening reduced the selection to 567 manuscripts. A final manual selection removed eight preprints that had not been peer-reviewed, 21 articles that used animal models, 27 articles that studied fetal alcohol spectrum disorders, 139 articles that did not use machine learning approaches, and 262 articles that did not primarily analyze alcohol consumption behaviors (e.g., withdrawal symptoms, alcohol-related liver diseases, drunk driving). This selection resulted in 110 original peer-reviewed human research studies^7^,^51^–^161^ that tested the model on data that it was not trained on, which is a key difference between machine learning and population-level statistical analysis.

## Results of the Literature Search

The search identified 2,618 articles for initial examination. Of those, 2,508 were excluded as described above, and 110 were included in the review. For each selected article, Appendix 1 records the number of subjects, mean age, sex ratio, prediction task (e.g., predicting relapse, AUD diagnosis classification), number of predictors, machine learning model used (e.g., random forest, SVM), and reported model accuracy (e.g., AUC and classification accuracy). If multiple alcohol-related prediction tasks were explored, the average prediction performance was recorded. If multiple machine learning models were explored, the model with the highest performance was recorded.

With respect to demographic factors, 85 of the 110 reviewed articles (or 77%) reported sex ratios: eight studies (9% of 85 articles) focused exclusively on men, 40 manuscripts (47%) were somewhat balanced between the sexes (i.e., the percentage of men was 40% to 60%), and three only studied women. Of the 110 reviewed articles, 89 manuscripts (81%) reported the mean age of cohorts, with most studies studying samples with a mean age from late childhood to middle-aged adulthood: specifically, 82 articles studied cohorts with mean ages of 10 to 50 years. Only seven studies focused on cohorts with a mean age greater than 50 years.

**Figure 2:**
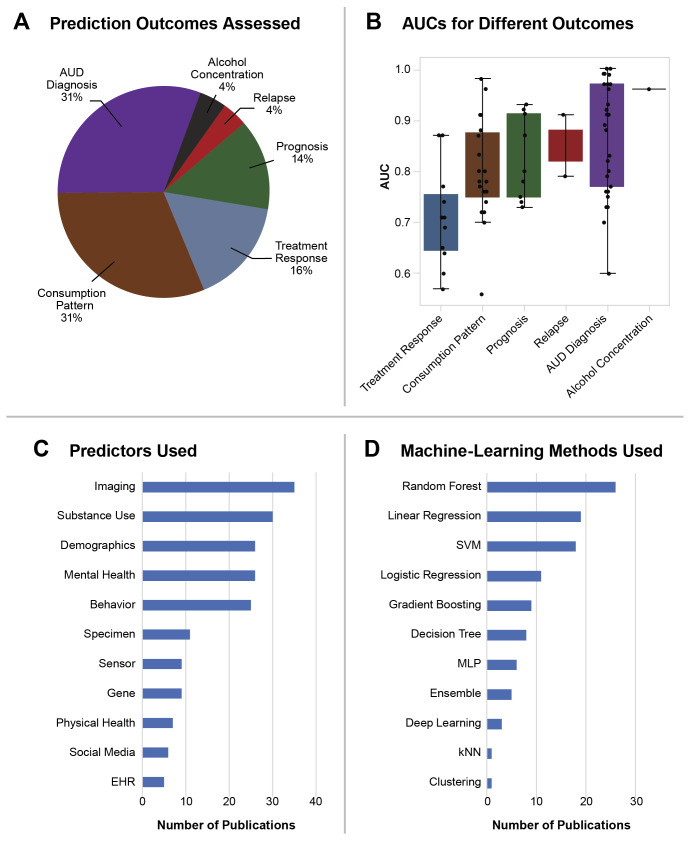
Characteristics of the studies identified by the review (A) Articles are categorized based on the clinical outcome targeted by the machine learning prediction: more than half the articles focus on predicting alcohol use disorder (AUD) diagnosis or consumption patterns. (B) Area under the curve (AUC) of the models of various outcomes. The most accurate clinical outcomes to predict were alcohol concentration and AUD diagnosis. (C) Number of studies that used a certain type of input predictors. The most common predictors were neuroimaging data, alcohol and other substance use patterns, demographics, mental health symptoms, and behavioral data from neuropsychological testing and self-reports. (D) Number of studies that used a certain type of machine learning model. The most frequently adopted models were random forest, linear regression, and support vector machine. *Note*. EHR, electronic health record; kNN, k-nearest neighbor; MLP, multilayer perceptron; SVM, support vector machine.

Among all prediction outcomes (Figure 2A), AUD diagnosis and alcohol consumption patterns were the two categories most often evaluated (both 31% of articles). Articles focusing on classifying AUD diagnosis reported a median AUC of 89% (Figure 2B), which was the highest reported among categories with at least two articles. In comparison, articles on predicting alcohol consumption had a median AUC of 78%.

Treatment response was the third most frequently predicted outcome but had the lowest median AUC (71%), followed by prognosis (14%, median AUC: 80%). Relapse and alcohol concentration both were assessed in only 4% of studies but had high median AUCs (85% and 96%, respectively). However, the very high AUC (96%) for sensor-based prediction of alcohol concentration requires confirmation as it was only published by one article.

**Figure 3:**
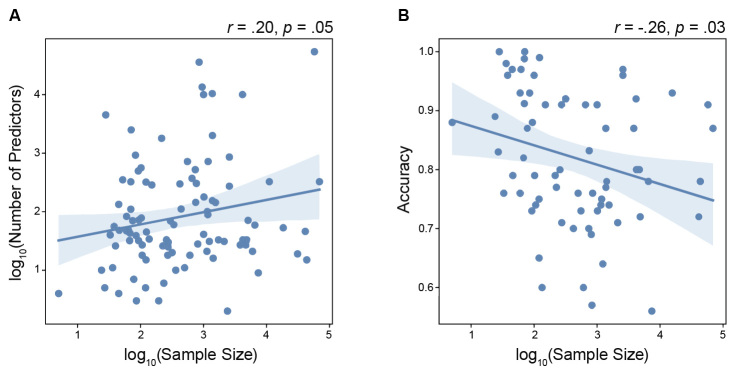
Correlation of sample size with number of predictors and accuracy The sample size showed (A) a positive correlation with the number of input features to the machine learning models; and (B) a negative correlation with model prediction accuracy.

Of the 110 articles, 46 (42%) used more than one type of predictor to predict outcomes. The most frequently used predictors were imaging data (32% of articles), followed by self-reported substance use (27%), demographics (24%), mental health (24%), and behavioral assessment (23%) (Figure 2C). Other data types were rarely used for prediction (10% or less). Conventional machine learning methods^162^ were the most popular approaches for prediction, including the random forest approach (used in 24% of 110 articles), linear regression (17%), and SVM (16%) (Figure 2D). Other approaches, such as logistic regression, gradient boosting, decision tree, multilayer perceptron, ensemble methods, k-nearest neighbor, and clustering were used in 10% of studies or less. Deep learning approaches were used in 3% of investigations. Studies with larger sample sizes tended to use higher-dimensional input features for prediction (i.e., used larger numbers of predictors) (Figure 3A; r = .20, p = .05); they also resulted in lower prediction accuracy (Figure 3B; r = −.26, p = .03).

## Results of the Studies Reviewed

### Demographics

Historically, AUD has been mostly diagnosed in men.^163^ However, in recent years, the prevalence of AUD among women has been rising. This is a concerning trend from a public health standpoint, as the adverse effects of alcohol misuse tend to be more severe in women than in men.^164^,^165^ This shift is evident in the articles reviewed herein, with only 9% of studies focusing exclusively on men, and the proportion of male participants in studies published since 2023 (*n* = 49) significantly lower (*p* = .032) compared to those published before 2023 (*n* = 36). Accordingly, the reviewed articles identified sex differences in the correlations of neural substrates and behavioral correlates with alcohol misuse and treatment response.^77^,^97^ For example, childhood trauma and microstructural integrity in temporal and motor structures were female-exclusive predictors of alcohol misuse in young adulthood, whereas social recognition ability, personality traits, and microstructural integrity in cerebellar, motor, and occipital structures were more important predictors for young males.^77^,^97^ Liver function test results predicted treatment response exclusively in males, whereas mental health symptoms were more informative predictors for females.^154^ Discovery of these distinctions was performed either by training a single machine learning algorithm on both sexes and comparing important predictors between males and females,^77^ or by training separate models for males and females and comparing the outcomes of the two models.^97^

In addition to sex, age is a critical demographic factor in alcohol use.^166^ Most recent machine learning studies have focused on adolescents through middle-aged adults, a critical window when drinking behaviors typically initiate^167^,^168^ and consolidate.^169^–^171^ As acknowledged in the reviewed articles, adolescence and early adulthood are critical for brain development^60^,^146^ and encompass major life transitions (e.g., transition from high school to college, entry into the workforce, and early parenthood). These transitions often involve shifts in social norms and heightened stress, both of which can drive alcohol use.^97^,^140^ Notably, binge drinking (defined as five or more drinks per occasion) peaks during the late teens to early 30s^172^ and is one of the strongest predictors of developing AUD later in life.^173^ By contrast, alcohol misuse in adulthood poses the greatest public health burden,^77^,^96^ including alcohol-related injuries (e.g., accidents, violence, fatalities)^174^–^176^ and economic costs (e.g., lost productivity and increased health care expenditure).^165^,^177^ Thus, machine learning studies are particularly valuable for simultaneously analyzing tens or hundreds of factors drawn from many conceptually different domains with the goal of identifying early markers of vulnerability and understanding later-life health and social outcomes to inform prevention and intervention strategies.

### Prediction Outcome

Distinguishing individuals with AUD from controls was a prevalent prediction task probed by machine learning analysis; it was addressed in 35 of the 110 articles (31%; Figure 2A). The high proportion may be due to the relatively high accuracy achieved by machine learning approaches (median AUC: 89.0%; Figure 2B). Predicting AUD is relatively simple compared to other clinical outcomes because it is a binary decision process based on clear diagnostic framing (i.e., meets the criteria of the Diagnostic and Statistical Manual of Mental Disorders: yes/no). Furthermore, AUD is typically associated with severe and enduring alterations in brain regions and behavior, as also documented by the reviewed articles.^84^,^129^ Accordingly, the prediction is often based on neuroimaging data (20 out of 35 articles). For example, prediction models based on structural and functional MRI have yielded converging evidence of AUD-related abnormalities in prefrontal circuits (executive dysfunction),^55^,^69^,^96^,^138^ ventral striatum (reward sensitivity),^84^,^96^ the cingulate cortex,^24^,^55^ default mode network,^138^,^139^ and the sensory cortex^96^,^107^ (Appendix 1). Although these regions also have been previously reported by literature based on traditional group-level analyses, the effect sizes revealed by machine learning approaches far exceed those identified by univariate statistical tests. For example, a neural network classifier achieved an AUC of 79% in classifying 51 people with AUD and 51 control subjects using whole-brain resting-state functional connectivity.^96^ In contrast, individual functional connectivity features typically exhibited group differences of t < 4.0, corresponding to an accuracy of only about 0.65.^96^

Predicting alcohol consumption patterns that did not meet the criteria for AUD was another task frequently investigated by machine learning models (35 studies, or 31% of the articles). Because alcohol misuse is among the strongest predictors of future AUD onset, this task holds significant value for prevention and policy design. Prediction models for this task often involved mixed data sources beyond imaging-based predictors. Common predictors of alcohol misuse identified by machine learning included sociodemographic factors (e.g., male sex or lower socioeconomic status),^51^,^119^,^130^,^142^ poor executive control,^51^,^119^ social behavior change,^83^,^119^ psychological dysfunction,^83^,^119^ and other substance use.^51^,^83^ The accuracy of these predictors in differentiating people who drink heavily from control subjects (median AUC 78%) was substantially higher than the accuracy of single predictors, which typically yielded only marginal improvements above chance.^51^,^61^,^119^ Collectively, these findings position machine learning approaches as a promising and potentially clinically actionable framework for early risk quantification and targeted prevention of AUD.

The third most predicted outcome of machine learning was treatment response. Common predictors identified by the reviewed articles included severity of dependence and craving at baseline,^7^,^98^,^127^ self-efficacy,^127^,^154^ and psychiatric comorbidity.^7^,^98^,^154^ Initial evidence also suggests that the quantitative, individualized prediction by machine learning models aggregating multiple predictors was more accurate than predictions made by human experts.^127^ Despite the potential, this prediction task was associated with the lowest median AUC (71%) among all prediction outcomes. One reason is that treatment response often is assessed using nonstandardized subjective measures (e.g., craving scales and self-reported relapse) that are known to exhibit low test-retest reliability and high individual variability.^71^ Further increasing variability was the wide difference in treatment duration, from a few days^132^ to a few years^136^ resulting in inconsistent timing of follow-up assessments. Thus, clear neurobiological and behavioral correlates with treatment outcomes remain elusive. In addition, studies on treatment outcomes also suffer from only a limited proportion of enrolled individuals completing the full course of treatment (e.g., 78% drop out rate^66^). This low completion rate contributes to significant class imbalance between individuals who do or do not respond to treatment. Collectively, these factors make it difficult to robustly train machine learning models and hinder their generalizability,^178^ which is reflected in treatment response emerging as the clinical outcome with the lowest average predictive accuracy (Figure 2B).

### Types of Data Used for Training Machine Learning Models

This review revealed that predictors of alcohol-related clinical outcomes encompass a diverse array of data types (Figure 2C), with the most popular one being medical imaging data. As mentioned, chronic heavy alcohol use results in significant volume loss in brain regions^55^ and disrupts brain function.^69^,^84^,^96^ Neuroimaging can capture these alterations noninvasively, making it appealing for investigating addiction mechanisms.^179^ Beyond improving the understanding of addiction mechanisms, the objectivity of nonfunctional neuroimaging transcends traditional self-report measures and thus has the potential for providing quantitative biomarkers for identifying individuals at elevated risk for drinking onset, predicting relapse,^180^ and assessing treatment outcomes.^181^

However, neuroimaging data also have some disadvantages. They are relatively expensive to acquire, can be susceptible to measurement variability and artifacts (e.g., head motion, scanner differences, and physiological fluctuations), and are associated with small effect sizes (typically explaining < 5% to 10% of the variance),^182^,^183^ because they are indirect proxies reflecting intermediate phenotypes (such as functional connectivity or activation patterns) rather than direct measures of behaviors, such as craving or relapse.^184^,^185^ In contrast to neuroimaging studies, neuropsychological assessments, self-reports, and demographic variables (e.g., alcohol use history, age of onset, or family history) are more proximally aligned with AUD outcomes and easier to collect, often translating to stronger predictive accuracy. This explains why the 18 studies using only neuroimaging as predictors had a lower median AUC of 79% than the 22 studies using a single type of nonneural predictors (median AUC: 91%).

No single data type (whether neuroimaging, genetic, behavioral, or self-report) fully captures the complexity of AUD and related clinical outcomes. In this review, 42% of studies investigated predictors from multiple modalities, which machine learning models are primed to do because their data-driven search does not require prior knowledge about the modalities they analyze. Each modality—whether it be genetic data, brain imaging, behavioral traits, or environmental exposures—generally captures a distinct yet complementary layer of risk or resilience.^26^,^128^,^186^ By seamlessly integrating these diverse information sources, the reviewed studies consistently indicated that multimodal machine learning models not only provide a more comprehensive representation of individual differences in alcohol-related outcomes but also enhance prediction accuracy and generalizability compared to single-modal models.^7^,^55^,^119^,^129^

## Discussion

### Model Design Choices

In general, the choice of machine learning models is heavily influenced by data set characteristics, particularly sample size and number of predictors, which are often strongly correlated (Figure 3A).^187^ Training highly complex models on a limited number of samples with many predictors can lead to “overfitting,” where the model captures noise patterns in the training data rather than generalizable relationships based on reliable measurements. Therefore, given that most alcohol-related datasets typically only include fewer than 1,000 subjects, conventional machine learning methods (i.e., random forest, SVM, linear regression)^162^ are more common because they depend on relatively fewer model parameters to learn. In contrast, state-of-the-art deep learning approaches require far larger data sets to generalize.^188^,^189^ Empirical studies have shown that these “sophisticated” models often have overestimated accuracy and are easily overfitted on small data sets.^189^

Moreover, the data in alcohol studies were often structured tabular data (e.g., demographic variables, self-report scores, or brain regional measurements) rather than raw, high-dimensional inputs (e.g., minimally processed structural MRI data). In these tabular-feature scenarios, traditional models often performed on par with or even better than deep learning because the key signals of the precomputed features (e.g., regional brain volumes or cognitive scores) and relatively simple prediction targets (often binary outcomes, such as diagnosis vs. control) did not require complex modeling. Recent evaluations on structured data sets found that tree-based ensembles (such as random forest and XGBoost^190^) consistently outperformed deep neural networks (which are considered the state-of-the-art in AI) for both purely numerical features and mixed data types.^191^,^192^ Thus, although deep learning has only begun to tread in alcohol research, its broader potential will depend on the availability of large, well-curated data sets.

Beyond prediction accuracy, the decision process of conventional models is generally easier to interpret compared with deep learning. This is essential for results from alcohol studies to be useful to clinicians and researchers who need to understand why a model makes a prediction to trust and act on it. For example, random forest can provide ranked feature importance^193^ to indicate which features are more influential to the model prediction. By contrast, deep neural networks are often regarded as “black boxes” with complex internal computations that defy straightforward explanations.^188^,^194^ This opacity can lead to reluctance in adopting deep learning models for medical decisions, because clinicians cannot easily verify what the model has learned or identify potential biases.^195^

Another important advantage of conventional machine learning methods over deep learning for alcohol researchers is the relatively low barrier to deployment and maintenance.^196^ Conventional methods are typically easier to set up, requiring fewer design choices and less parameter tuning than deep learning approaches. They handle mixed data types and occasional missing values with minimal preprocessing, whereas deep networks usually demand careful data cleaning and customization for each new task. Conventional machine learning algorithms also run efficiently on standard computational resources, with training of the models often completed in seconds or minutes on a normal personal computer. In contrast, deep learning approaches necessitate high-performance GPUs or cloud infrastructure, which is expensive and harder to operate.^194^

In summary, the dominance of conventional machine learning approaches in alcohol-related neuroimaging and behavioral prediction can be attributed to a combination of data limitations, interpretability needs, and ease of use. Although this observation might be biased by the search criteria of this review, which were confined to nonspecific key words, such as “machine learning” and “deep learning,” none of the deep learning approaches used in the studies were based on state-of-the-art architectures (e.g., transformers, generative models, and large language models) that recently have been the focus of public discourse.

### Key Methodological Challenges and Future Directions for AI in Alcohol Research

Despite the increased use of machine learning analyses in alcohol research, several methodological limitations continue to constrain the full potential of this technology. The limiting factor most commonly mentioned among the reviewed articles is the number of available samples (25 articles). As mentioned, generalizability of machine learning methods relies on training them on heterogeneous data sets, which requires collecting data from a large number of samples. Funding institutions have identified this need and have recently funded several large, multicenter studies, such as NCANDA, ABCD, COGA, and MTF. However, simply increasing sample size does not overcome existing limitations; machine learning methodology also needs to be tailored to study such large samples in alcohol research. This review revealed a negative correlation between sample size and model prediction accuracy (Figure 3B), contradicting the general understanding that the accuracy of machine learning should increase with the size of training data. This paradox reflects a broader challenge in psychiatry increasingly recognized in the literature,^197^,^198^ namely the trade-off between predictive accuracy and population heterogeneity.^197^ Smaller data sets tend to be more homogeneous, allowing models to learn a single predictive pattern with high accuracy. However, this pattern is likely specific to a restricted subpopulation, thus resulting in a lower accuracy in larger heterogenous data sets; this was pointed out by 35 articles of this review. A promising way forward lies in the development of foundation models.^199^ These are large-scale, general-purpose models that are first trained on broader data sets, including those not necessarily related to alcohol research. Once pretrained, these models can be fine-tuned to specific subpopulations or clinical questions (e.g., predicting relapse in young females or treatment response in people with chronic AUD with liver disease). Creating such models could be essential for moving from subjective, population-level generalizations toward truly individualized, quantitative, and context-sensitive predictions that are the foundational promise of precision psychiatry.

Another challenge in using machine learning to advance alcohol research is the modeling of confounders, which were stringently accounted for by only 27 of the 110 articles (25%) reviewed. When confounding variables (e.g., age, sex, or comorbid mental health conditions) influence both the input features (e.g., neural or behavioral measures) and the outcome (e.g., alcohol consumption or treatment response), models may learn spurious associations that lead to misinterpretation of machine learning findings^200^ as also acknowledged by 24 articles of this review. For instance, age affects both brain connectivity and drinking patterns.^201^ If age is not adjusted for, the model may “predict” alcohol misuse by detecting brain maturation, not alcohol effects. Similarly, alcohol consumption patterns significantly differ between males and females; consequently, a machine learning model not controlling for sex might simply detect sex differences in the input predictors. Another type of confound is caused by comorbidity (mentioned by nine articles), because alcohol use often co-occurs with other physical and psychiatric symptoms (e.g., liver disease and depression) and other substance use. The identified predictors hence might not be linked to alcohol outcomes but to other confounding phenotypes. This type of signal leakage caused by confounds^202^,^203^ often results in misleading model interpretations^204^ and inflates accuracy scores (e.g., AUC, accuracy). This is especially problematic in nonrandomized, observational data sets typically encountered in alcohol research.

Mitigation of confounding effects is well established in traditional statistics but often underdeveloped or overlooked in machine learning research, which has historically only focused on maximizing predictive accuracy. In particular, modern deep learning models have the capacity to encode complex nonlinear confounding effects that are hard to detect or adjust for.^203^ This gap could be addressed by treating modeling confounders as a core component of the machine learning pipeline. Researchers could apply appropriate preprocessing techniques (e.g., residualization, stratification, or harmonization^205^) before training, and may want to consider models specifically designed to reduce confounding, such as domain-invariant^206^ or confounder-aware approaches.^61^,^207^,^208^ Also, evaluation of the models could be enhanced by going beyond reporting a single AUC and incorporating fairness metrics^209^ (e.g., subgroup AUCs) and sensitivity analyses^210^ (e.g., performance changes with and without confounders). Validation of models on external data sets where the distribution of individual confounders differs would be crucial for ensuring generalizability and robustness.

Beyond modeling confounding effects, six of the 110 articles reviewed here explicitly mentioned the limitation that the adopted machine learning approach could only reveal statistical association between predictors and outcome but could not reveal causality between them. This issue highlights an urgent need for a paradigm shift in machine learning applications within alcohol research. Rather than focusing solely on optimizing prediction accuracy, models could also be designed to reveal dependencies and potential causal pathways among variables. New models designed to discern how external environmental and sociodemographic factors (i.e., those that give rise to population heterogeneity) moderate genetic, neural, and behavioral underpinnings of AUD in a data-driven fashion could move the field forward.^211^,^212^ A promising future direction is the application of canonical correlation analysis^213^ and its variants, which can uncover shared patterns across domains that are not only interpretable but can also serve as input features for downstream phenotype prediction.^214^ To further capture directional influences, structural equation modeling^215^ can be integrated to model hypothesized causal pathways and test how upstream factors (e.g., genes, environment) propagate their effects through intermediate phenotypes (e.g., brain function) to shape behavioral outcomes. This causal approach might offer a powerful new avenue in generative modeling^212^ that could ultimately enable virtual interventions, allowing researchers to test “what-if” scenarios without carrying out real-world in vivo experiments. For example, a generative model could simulate how removing environmental risk, improving modifiable behaviors, such as sleep hygiene, or neural simulation could predict downstream effects on alcohol-related behaviors. Achieving this vision will require alcohol researchers to closely work together with AI methodologists and implementation scientists, such as facilitated by Stanford’s AI for Mental Health Initiative.^216^

As mentioned, compared to statistical group analysis that only quantifies a population average trend, a strength of machine learning is to condense multivariate patterns across many features to an individualized score that can be mapped directly onto clinical decisions (e.g., risk of AUD onset or likelihood of relapse). However, none of the reviewed studies discussed the deployment of machine learning approaches in clinical settings. This issue is symptomatic for psychiatry in general, whereas other areas of medicine are beginning to integrate AI models into clinical workflows and even clinical trials.^217^,^218^ Unlike other fields of medicine that rely on measurable physiological indicators, psychiatry still relies heavily on subjective reports and assessment for diagnosis and treatment. This lack of objective, quantifiable targets poses a major barrier to training and validating clinically actionable machine learning models. Consequently, as of March 2026, none of the FDA-approved AI-enabled medical devices were tailored toward psychiatric conditions.^219^,^220^ Unique to the field of alcohol research is that studies soon will be able to replace self-reported alcohol use with real-time quantitative measurements provided by noninvasive alcohol biosensors.^221^,^222^ Such real-time, quantitative monitoring of alcohol use will provide objective, continuous data that can transform model development and clinical validation.

To fully realize this potential, rigorous evaluation standards must be established. The studies reviewed exhibit substantial variability in validation practices: thus, 18% of studies relied on a single train/test split without cross-validating results on all samples, only 12% of studies validated findings on external data sets, and 16% of articles adopted potentially flawed methodological designs, such as double dipping (e.g., feature selection on the full data set before training) and data leakage (e.g., information of test samples were used for training). The establishment of standardized benchmark data sets and data splits, as well as the preregistration of machine learning analysis and evaluation plans,^223^ including training and testing data construction, can help remedy these concerns. Once quantitative assessments are coupled with stringent model evaluation, researchers will be able to easily test the validity of their models, allowing the field to gain a deeper insight into addiction. Ensuring the responsible deployment of this technology in clinical settings will enable practitioners to diagnose patients based on quantitative markers and objectively assess the progress of alcohol treatments. Finally, prevention programs might be able to accurately determine risk of alcohol misuse in individuals. By doing so, the field of alcohol research could be a trailblazer in psychiatry as it would use AI technology to radically improve the diagnosis and treatment of a psychiatric disease, namely AUD.

## Conclusion

This review focused on studies employing machine learning methods that, in contrast to hypothesis-driven analyses, can uncover complex multivariate patterns predictive of alcohol-related outcomes on an individual basis. Most of the 110 reviewed articles trained conventional machine learning approaches on a single data modality to predict alcohol consumption or AUD diagnosis in cohorts with mean ages between 12 and 50 years.

Studies were limited by the small number of available samples, not being able to gain insights about causality, and failing to account for confounders. Significantly improving the diagnosis and treatment of AUD will require harnessing the full potential of recent advances in deep learning (such as foundation models).^224^ Specifically, fostering multidisciplinary research teams to create rigorous and trustworthy models can help analyze large, multimodal data sets that can capture the multifaceted nature of alcohol use and its comorbidities based on quantitative measures. Additionally, setting guidelines for the responsible development and deployment of these machine learning models would help ensure that these approaches will improve precision alcohol treatment.

## Figures and Tables

**Figure 1:**
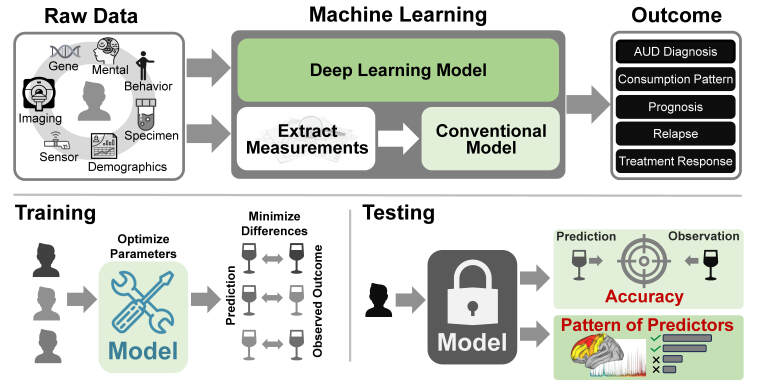
Machine learning methods can transform complex, multimodal data into individualized predictions of alcohol use disorder (AUD) and related outcomes Data modalities used by studies reviewed here included, among others, behavior, biological specimens, demographics, sensor data, neuroimaging, genes, and mental health assessments. Raw data were either directly analyzed by deep learning models or first extracted into aggregate measurements for use in conventional machine learning approaches, which were used in most studies reviewed. AUD-related outcomes predicted by these methods encompassed AUD diagnosis, consumption patterns, prognosis, relapse, and treatment response. To obtain the predictions, the studies first trained the machine learning models by determining the parameter setting that minimized the difference between predicted and observed outcomes on the training data set. The studies then measured the accuracy of the predictions and the constellation of measurements (i.e., pattern) that drove the predictions, on a separate test set.

**Appendix 1:** Characteristics of the 110 Peer-Reviewed Human Studies Identified in This Article

Author	Title	AI Model	Sample Size	Predictor Category	Number of Predictor Variables	Area Under the Curve	External Data Use	Mean Age (Years)	Male Proportion
***Alcohol Concentration***									
Fairbairn (2025)^57^	A wearable alcohol biosensor: exploring the accuracy of transdermal drinking detection	Ensemble method	100	Sensor		96%	No	24.2	50%
Oszkinat (2022)^62^	An abstract parabolic system-based physics-informed long short-term memory network for estimating breath alcohol concentration from transdermal alcohol biosensor data	LSTM	40	Sensor	26		No	27	50%
Li (2021)^88^	Estimation of blood alcohol concentration from smartphone gait data using neural networks	Deep learning	65	Sensor	48		No	30.8	38%
Ariss (2023)^89^	Examining new-generation transdermal alcohol biosensor performance across laboratory and field contexts	ExtraTrees	256	Sensor			Yes	25.5	45%
***AUD Diagnosis***									
Guggenmos (2020)^55^	A multimodal neuroimaging classifier for alcohol dependence	Ensemble method	216	Neuroimaging	1,791	79%	No	45	16%
Mulholland (2023)^59^	Adapter protein complex 2 in the orbitofrontal cortex predicts alcohol use disorder	sPLS-DA	28	Specimen	4,503	100%	No	55	50%
Anuragi (2019)^114^	Alcohol use disorder detection using EEG Signal features and flexible analytic wavelet transform	SVM	122	Neuroimaging	15	99%	No		
Mumtaz (2017)^64^	An EEG-based machine learning method to screen alcohol use disorder	Logistic regression	45	Neuroimaging	4		No	51.1	
Zhang (2022)^67^	Association between abnormal plasma metabolism and brain atrophy in alcohol-dependent patients	XGBoost	226	Specimen	26	77%	No	40	100%
Komarnyckyj (2022)^68^	At-risk alcohol users have disrupted valence discrimination during reward anticipation	Multivariate discriminant analysis	44	Neuroimaging			No	23.8	54%
Song (2024)^69^	Atypical effective connectivity from the frontal cortex to striatum in alcohol use disorder	Uncertain	62	Neuroimaging		97%	No	38	100%
Mumtaz (2016)^161^	Automatic diagnosis of alcohol use disorder using EEG features	Logistic model trees	45	Neuroimaging	133	97%	No	49	
Besong (2024)^70^	Brain lncRNA-mRNA co-expression regulatory networks and alcohol use disorder	Random forest	24	Gene	10	89%	No	48.4	50%
Zhu (2021)^74^	Combining metabolomics and interpretable machine learning to reveal plasma metabolic profiling and biological correlates of alcohol-dependent inpatients: What about tryptophan metabolism regulation?	Decision tree	85	Specimen	39	93%	No	45.5	100%
Ebrahimi (2021)^78^	Deep neural network to identify patients with alcohol use disorder	MLP	2,571	EHR	857	97%	No		
Kamarajan (2022)^84^	Differentiating individuals with and without alcohol use disorder using resting-state fMRI functional connectivity of reward network, neuropsychological performance, and impulsivity measures	Random forest	60	Neuroimaging, behavior	47	93%	No	34	100%
Chen (2020)^85^	Discrimination of alcohol dependence based on the convolutional neural network	Deep learning	317	Gene, demographic	20	92%	No	40	
Sangle (2025)^113^	Explaining electroencephalogram channel and subband sensitivity for alcoholism detection	Neural network	122	Neuroimaging	320	99%	No	35.8	
Ebrahimi (2022)^94^	Identification of clinical factors related to prediction of alcohol use disorder from electronic health records using feature selection methods	Random forest	2,571	EHR	272	96%	No	70	47%
Vergara (2022)^96^	Identifying alcohol use disorder with resting state functional magnetic resonance imaging data: A comparison among machine learning classifiers	SVM, MLP, logistic regression, random forest	102	Neuroimaging	561	79%	No	34.6	65%
Guleken (2025)^99^	Investigating the Impact of long-term alcohol consumption on serum chemical changes: Fourier-transform infrared spectroscopy for human blood serum	SVM	71	Specimen	2,493	100%	No		
Morris (2016)^101^	Jumping the gun: mapping neural correlates of waiting impulsivity and relevance across alcohol misuse	SVM	134	Neuroimaging		60%	No	32.4	53%
Zhang (2025)^105^	Machine learning models for diagnosis and risk prediction in eating disorders, depression, and alcohol use disorder	Elastic net	258	Behavior, environment, mental health, substance use, physical	33	80%	Yes	22.6	42%
Zhu (2024)^107^	Machine learning of functional connectivity to biotype alcohol and nicotine use disorders	MLP	850	Neuroimaging	35,778	76%	No	54	72%
Kummerfeld (2018)^112^	Methodological advances in the study of hidden variables: A demonstration on clinical alcohol use disorder data	GFCI	362	Substance use, mental health	10		No		
Ruiz-España (2023)^159^	MRI texture-based radiomics analysis for the identification of altered functional networks in alcoholic patients and animal model	SVM	68	Neuroimaging	43	82%	Yes	43.9	100%
Lee (2024)^117^	Multimodal-based machine learning approach to classify features of Internet gaming disorder and alcohol use disorder: A sensor-level and source-level resting-state electroencephalography activity and neuropsychological study	Logistic regression	124	Neuroimaging, behavior, mental health, demographic	45		No	26.4	82%
Adeli (2019)^160^	Novel machine learning identifies brain patterns distinguishing diagnostic membership of human immunodeficiency virus, alcoholism, and their comorbidity of individuals	Logistic regression	421	Neuroimaging	298	70%	No	47.5	62.4%
Mohd Nazri (2025)^121^	Partial directed coherence analysis of resting-state EEG signals for alcohol use disorder detection using machine learning	SVM	70	Neuroimaging	324	98.8%	No	48	56%
Yang (2022)^125^	Polygenic risk prediction based on singular value decomposition with applications to alcohol use disorder	Linear regression	11,982	Gene			Yes		
Kamarajan (2023)^129^	Predicting alcohol-related memory problems in older adults: A machine learning study with multidomain features	Random forest	94	Neuroimaging, behavior, alcohol use, gene	72	88%	No	40	55%
Kinreich (2021)^7^	Predicting risk for alcohol use disorder using longitudinal data with multimodal biomarkers and family history: A machine learning study	SVM	656	Neuroimaging, substance use, gene	371	91%	No		57%
Zhu (2018)^138^	Random forest based classification of alcohol dependence patients and healthy controls using resting state MRI	Random forest	92	Neuroimaging	496	73%	No	36	64%
Kamarajan (2020)^139^	Random forest classification of alcohol use disorder using fMRI functional connectivity, neuropsychological functioning, and impulsivity measures	Random forest	60	Neuroimaging, behavior	83	76%	No	34	100%
Rosato (2019)^143^	Salivary microRNAs identified by small RNA sequencing and machine learning as potential biomarkers of alcohol dependence	Random forest	120	Gene	5	75%	No	41	46%
Dagnew (2024)^147^	Toward AI-driven neuro-epigenetic imaging biomarker for alcohol use disorder: A proof-of-concept study	SVM	27	Neuroimaging	5	83%	No	38.8	70%
Lee (2019)^151^	Using machine learning to classify individuals with alcohol use disorder based on treatment seeking status	Decision tree	1,014	Environment, behavior, mental health, specimen	178	73%	Yes	43.3	70.6%
To (2020)^155^	Validation of an alcohol misuse classifier in hospitalized patients	LASSO	1,000	EHR	10,000	91%	No		49
***Consumption Pattern***									
O’Halloran (2018)^51^	A combination of impulsivity subdomains predict alcohol intoxication frequency	Elastic net	106	Substance use, behavior	18		No	19.5	56%
Kim (2021)^52^	A deep learning algorithm to predict hazardous drinkers and the severity of alcohol-related problems using K-NHANES	MLP	69,187	Physical, specimen	325	87%	No		
Bonnell (2020)^53^	A machine learning approach to identification of unhealthy drinking	Random forest	43,545	Demographic, specimen	15	78%	No	49	48.6%
Johnson (2024)^54^	A machine learning model for the prediction of unhealthy alcohol use among women of childbearing age in Alabama	SVM	2,397	Demographic, mental health	2		No	30.6	0%
Kumari (2022)^158^	A novel method for predicting time of alcohol use based on personality traits and demographic information	Random forest		Behavior, demographic		77%			
May (2022)^56^	A prospective investigation of youth alcohol experimentation and reward responsivity in the ABCD study	SVM, linear regression	7,409	Neuroimaging, substance use	9	56%	No	10	50%
Sun (2023)^60^	Adolescent alcohol use is linked to disruptions in age-appropriate cortical thinning: an unsupervised machine learning approach	NMF	657	Neuroimaging			No	15.6	50%
Park (2018)^61^	Alcohol use effects on adolescent brain development revealed by simultaneously removing confounding factors, identifying morphometric patterns, and classifying individuals	Linear regression	750	Neuroimaging	144	83.2%	No	15.8	47%
Curtis (2018)^71^	Can Twitter be used to predict county excessive alcohol consumption rates?	Ridge regression	1,384	Social media	2,000		No		
Stevely (2021)^73^	Combinations of drinking occasion characteristics associated with units of alcohol consumed among British adults: an event-level decision tree modeling study	Linear regression, decision tree	18,409	Substance use, behavior	53		No		
Li (2025)^77^	Connectomics modeling of regional networks of white-matter fractional anisotropy to predict the severity of young adult drinking	Linear regression	949	Neuroimaging	13,456		No	28.8	48%
Crocamo (2020)^80^	Detecting binge drinking and alcohol-related risky behaviors from Twitter’s users: an exploratory content- and topology-based analysis	SVM	500	Social media	11	76%	No		
Bush (2024)^82^	Development of an accelerometer-based wearable sensor approach for alcohol consumption detection	Random forest	194	Sensor	3		No	37.1	33%
Lee (2025)^83^	Development of deep learning auto-encoder algorithms for predicting alcohol use in Korean adolescents based on cross-sectional data	MLP	41,239	Physical, behavior, environment	46	72%	No	15	57%
Liang (2021)^86^	DNA methylation signature on phosphatidylethanol, not on self-reported alcohol consumption, predicts hazardous alcohol consumption in two distinct populations	Elastic net	1,549	Gene	143	74%	Yes	42	79%
Marengo (2019)^90^	Exploring the association between problem drinking and language use on Facebook in young adults	Random forest	296	Social media	69		No	28.4	33%
Lin (2022)^91^	External validation of a machine learning classifier to identify unhealthy alcohol use in hospitalized patients	Logistic regression	57,605	EHR	54,000	91%	Yes	61	42%
Huang (2017)^93^	High-resolution temporal representations of alcohol and tobacco behaviors from social media data	Logistic regression		Social media		80%	No		
Zhu (2022)^95^	Identifying alcohol misuse biotypes from neural connectivity markers and concurrent genetic associations	MLP	739	Neuroimaging	521	70%	No	28.5	43%
Sania (2025)^102^	K-nearest neighbor algorithm for imputing missing longitudinal prenatal alcohol data	kNN	1,1083	Substance use	325		No		0%
Rezapour (2023)^109^	Machine learning-based analytics of the impact of the Covid-19 pandemic on alcohol consumption habit changes among U.S. healthcare workers	XGBoost	273	Mental health, behavior	25	91%	No	38	30%
Bae (2018)^115^	Mobile phone sensors and supervised machine learning to identify alcohol use events in young adults: implications for just-in-time adaptive interventions	Random forest	38	Sensor	56	96%	No	23	60%
Grodin (2021)^116^	Modeling motivation for alcohol in humans using traditional and machine learning approaches	Random forest	67	Demographic, substance use, behavior	32		No	29	54%
Afshar (2019)^118^	Natural language processing and machine learning to identify alcohol misuse from the electronic health record in trauma patients: development and internal validation	Logistic regression	1,422	EHR	16	78%	No	44	70%
Squeglia (2017)^119^	Neural predictors of initiating alcohol use during adolescence	Random forest	137	Neuroimaging, demographic, mental health, behavior	34		No	13	55%
Marcon (2021)^123^	Patterns of high-risk drinking among medical students: A web-based survey with machine learning	Elastic net	4,840	Demographic, environment, substance use	33	72%	No	21.8	24%
Soyster (2022)^126^	Pooled and person-specific machine learning models for predicting future alcohol consumption, craving, and wanting to drink: A demonstration of parallel utility	Elastic net	33	Behavior, mental health, substance use	40	76%	No	19	7%
Leaks (2023)^130^	Predicting moderate drinking behaviors in National Health and Nutrition Examination Survey participants using biochemical and demographical factors with machine learning	Ensemble method	4,219	Demographic, specimen	33	80%	No	51.9	43%
Agarwal (2025)^134^	Prediction of alcohol intake patterns with olfactory and gustatory brain connectivity networks	Linear regression	1,003	Neuroimaging	41		No	28.7	54%
Schwebel (2024)^140^	Regression tree applications to studying alcohol-related problems among college students	Decision tree	5,090	Demographic, substance use, behavior, mental health	71		Yes	20.8	29%
Duadi (2025)^141^	Remote sensing of alcohol consumption using machine learning speckle pattern analysis	XGBoost	5	Sensor	4	88%	No	35.2	30%
Fede (2019)^142^	Resting state connectivity best predicts alcohol use severity in moderate to heavy alcohol users	Random forest	83	Neuroimaging, demographic	922		No	41	63%
Didier (2024)^144^	Signal processing and machine learning with transdermal alcohol concentration to predict natural environment alcohol consumption	Elastic net	36	Sensor	11	98%	No	27.3	50%
Foster (2022)^157^	Young Swiss men’s risky single-occasion drinking: identifying those who do not respond to stricter alcohol policy environments	Random forest	5,986	Demographic, mental health, behavior, environment	21		No	20	100%
***Prognostic***									
Amialchuk (2021)^150^	Applying machine learning methods to model social interactions in alcohol consumption among adolescents	Gradient boosting	4,686	Demographic, substance use	27	80%	No	14.9	48%
Uceta (2024)^72^	Clustering electrophysiological predisposition to binge drinking: an unsupervised machine learning analysis	Clustering	103	Neuroimaging	78		No	13.7	48%
Pinar-Sanchez (2022)^76^	Common laboratory parameters are useful for screening for alcohol use disorder: designing a predictive model using machine learning	Naive bayes	337	Specimen	60		No	44	75%
Miranda (2024)^79^	DeepBiomarker2: prediction of alcohol and substance use disorder risk in post-traumatic stress disorder patients using electronic medical records and multiple social determinants of health	Deep learning	15,612	Environment, behavior, mental health		93%	No	36	27%
Bharat (2023)^81^	Development and evaluation of a risk algorithm predicting alcohol dependence after early onset of regular alcohol use	Ensemble method	6,526	Demographic, substance use, mental health, environment	59	78%	No	15	50%
Zhao (2024)^97^	Identifying high school risk factors that forecast heavy drinking onset in understudied young adults	SVM	106	Mental health, behavior, environment	27	74%	No	16.1	41%
Ruberu (2021)^100^	Joint risk prediction for hazardous use of alcohol, cannabis, and tobacco among adolescents: A preliminary study using statistical and machine learning	Lasso regression	270	Demographic, environment, substance use	18		No	15.5	74%
Andrade (2024)^103^	Large-scale longitudinal analysis of the progression of alcohol use among members of a social media platform: an observational study	Random forest	4,160	Social media	10,006	92%	No		
Bae (2023)^104^	Leveraging mobile phone sensors, machine learning, and explainable artificial intelligence to predict imminent same-day binge-drinking events to support just-in-time adaptive interventions: algorithm development and validation study	XGBoost	75	Sensor	70		No	22.4	29%
Afzali (2019)^110^	Machine-learning prediction of adolescent alcohol use: a cross-study, cross-cultural validation	Elastic net	3,826	Demographics, mental health, behavior	27	87%	Yes	12.8	50.8%
Leenaerts (2024)^124^	Person-specific and pooled prediction models for binge eating, alcohol use and binge drinking in bulimia nervosa and alcohol use disorder	Elastic net	70	Environment, social, behavior, mental health, substance use	110	91.2%	No	21	0
Chung (2024)^135^	Prediction rules identify which young adults have higher rates of heavy episodic drinking after exposure to 12-week text message interventions	Ensemble method	1,131	Substance use	21		No	22.1	32%
Rane (2022)^145^	Structural differences in adolescent brains can predict alcohol misuse	SVM, logistic regression, gradient boosting	1,182	Neuroimaging	719	75%	No	14	47%
Weidacker (2022)^146^	The prediction of resilience to alcohol consumption in youths: insular and subcallosal cingulate myeloarchitecture	RVR	86	Neuroimaging	3		No	21.76	67%
Rane (2023)^148^	Uncontrolled eating and sensation-seeking partially explain the prediction of future binge drinking from adolescent brain structure	SVM	555	Neuroimaging	719	73%	No	28	47%
***Relapse***									
Lin (2020)^63^	An analysis of the effect of mu-opioid receptor gene (OPRM1) promoter region DNA methylation on the response of naltrexone treatment of alcohol dependence	Random forest	93	Gene	32		No	50	100%
Wyant (2024)^106^	Machine learning models for temporally precise lapse prediction in alcohol use disorder	XGBoost	151	Substance use, mental health, behavior	286	91%	No	41	51%
Sekutowicz (2019)^120^	Neural response patterns during Pavlovian-to-instrumental transfer predict alcohol relapse and young adult drinking	SVM	52	Neuroimaging	350		No	44.5	27%
Seo (2015)^133^	Predicting the future relapse of alcohol-dependent patients from structural and functional brain images	RSLVQ	46	Neuroimaging	48	79%	No	40.7	65%
Schwebel (2022)^152^	Using machine learning to examine predictors of treatment goal change among individuals seeking treatment for alcohol use disorder	Random forest, decision tree	441	Demographic, substance use, mental health, environment	111		No	34.5	58.3%
***Treatment Response***									
Smink (2021)^65^	Analysis of the emails from the Dutch web-based intervention “Alcohol de Baas”: assessment of early indications of drop-out in an online alcohol abuse intervention	Decision tree	770	Social media	304		No	46	44%
Collin (2024)^66^	Analyzing dropout in alcohol recovery programs: A machine learning approach	SVM	31,087	Mental health	19		Yes		82.7%
Derksen (2025)^87^	Effectiveness of machine learning-based adjustments to an eHealth intervention targeting mild alcohol use	Random forest	234	Behavior	6		No	58.2	
Schwebel (2025)^92^	Finding purpose: integrated latent profile and machine learning analyses identify purpose in life as an important predictor of high-functioning recovery after alcohol treatment	Random forest	809	Mental health, physical health, environment, alcohol use, other substance use, demographics	28	69%	No	40.3	70%
Cavicchioli (2021)^98^	Investigating predictive factors of dialectical behavior therapy skills training efficacy for alcohol and concurrent substance use disorders: A machine learning study	Elastic net	275	Substance use, mental health	30	71%	No	47	59%
Symons (2019)^108^	Machine learning vs. addiction therapists: A pilot study predicting alcohol dependence treatment outcome from patient data in behavior therapy with adjunctive medication	Decision tree	830	Demographic, substance use, mental health		57%	Yes	41	
Hinton (2017)^111^	Metabolomics biomarkers to predict acamprosate treatment response in alcohol-dependent subjects	Lasso regression	120	Demographic, mental health, substance use, specimen		65%	No	45	68%
Witkiewitz (2023)^122^	Patterns of drinking behavior around a treatment episode for alcohol use disorder: predictions from pretreatment measures	XGBoost	1,726	Demographic, substance use, behavior, physical, mental health, environment	33		Yes	20	75%
Symons (2020)^127^	Predicting alcohol dependence treatment outcomes: a prospective comparative study of clinical psychologists vs. “trained” machine learning models	Logistic regression	1,236	Demographic, substance use	31	64%	Yes	40	66%
Kinreich (2021)^128^	Predicting alcohol use disorder remission: a longitudinal multimodal multifeatured machine learning approach	SVM	1,376	Neuroimaging, gene, demographic	10,391	87%	No	33	60%
Zhang (2022)^131^	Predicting Readmission following hospital treatment for patients with alcohol related diagnoses in an Australian regional health district	SVM, random forest					No		
Ramos (2021)^132^	Predicting success of a digital self-help intervention for alcohol and substance use with machine learning	Random forest	2,126	Behavior	31	71%	No		
Gueorguieva (2015)^136^	Predictors of abstinence from heavy drinking during follow-up in COMBINE	Logistic regression	1,150	Demographic, substance use, behavior, physical	100	74%	No		
Wallach (2022)^137^	Predictors of abstinence, no heavy drinking days, and a 2-level reduction in World Health Organization drinking levels during treatment for alcohol use disorder in the COMBINE study	Decision tree	1,168	Substance use, physical, demographic, specimen, mental health	89		No		
Lindner (2020)^149^	Using alcohol consumption diary data from an Internet intervention for outcome and predictive modeling: a validation and machine learning study	Random forest	607	Substance use	18	60%	No		
Walters (2021)^153^	Using machine learning to identify predictors of imminent drinking and create tailored messages for at-risk drinkers experiencing homelessness	Gradient boosting	78	Behavior	7	87%	No	46.2	84%
Roberts (2022)^154^	Using machine learning to predict heavy drinking during outpatient alcohol treatment	Random forest	1,383	Demographic, substance use, mental health	154	77%	No	44	69%
Rehm (2015)^156^	Who receives treatment for alcohol use disorders in the European Union? A cross-sectional representative study in primary and specialized health care	Logistic regression	8,476	Substance use, mental health			No		

*Note*. ABCD, Adolescent Brain Cognitive Development; AI, artificial intelligence; AUD, alcohol use disorder; EHR, electronic health record; GFCI, greedy fast causal inference; kNN, k-nearest neighbor; LASSO, least absolute shrinkage and selection operator; LSTM, long short-term memory; MLP, multilayer perceptron; MRI, magnetic resonance imaging; NMF, nonnegative matrix factorization; RSLVQ, robust soft learning vector quantization; RVR, relevant vector regression; sPLS-DA, sparse partial least squares discriminant analysis; SVM, support vector machine.
